# Association between serum homocysteine level and cognitive function in middle-aged type 2 diabetes mellitus patients

**DOI:** 10.1371/journal.pone.0224611

**Published:** 2019-11-06

**Authors:** Johanda Damanik, Andre Mayza, Andhika Rachman, Rani Sauriasari, Melly Kristanti, Putri Syahida Agustina, Alexander Randy Angianto, Pukovisa Prawiroharjo, Em Yunir

**Affiliations:** 1 Department of Internal Medicine, Faculty of Medicine Universitas Indonesia – Cipto Mangunkusumo National General Hospital, Jakarta, Indonesia; 2 Department of Neurology, Faculty of Medicine Universitas Indonesia – Cipto Mangunkusumo National General Hospital, Jakarta, Indonesia; 3 Department of Internal Medicine, Division of Haematology and Medical Oncology, Faculty of Medicine Universitas Indonesia – Cipto Mangunkusumo National General Hospital, Jakarta, Indonesia; 4 Faculty of Pharmacy, Universitas Indonesia, Jakarta, Indonesia; 5 Department of Internal Medicine, Division of Endocrinology and Metabolism, Faculty of Medicine Universitas Indonesia – Cipto Mangunkusumo National General Hospital, Jakarta, Indonesia; Nathan S Kline Institute, UNITED STATES

## Abstract

Type-2 diabetes mellitus (T2DM) is strongly associated with various complications, including cognitive impairment. Diabetic complication is related with structural and functional changes of brain. Studies investigated that homocysteine as an independent risk factor of several organ complications. This marker might have a role in pathogenesis of cognitive impairment in T2DM patients. We aimed to know the association between serum homocysteine level and cognitive impairment in middle-aged T2DM populations. The study was a cross-sectional study involving 97 T2DM patients aged <60 years old. Cognitive assessment was based on validated Indonesian version of Montreal Cognitive Assessment (MoCA-INA) test. Besides, serum homocysteine level (Hcy) was measured based on standard laboratory assay. Filling out the questionnaire of MoCA-INA was conducted when patients came to take the blood sample. This study used independent t-test, chi-square and multivariate logistic regression model to analyze the data. There were 47 subjects (48.5%) with mild cognitive impairment (MCI). Delayed recall was the most impaired domain (94.8%). There was no significant mean difference of serum Hcy level in MCI and non-MCI group (11.99±3.27 μmol/L vs 12.36±4.07 μmol/L respectively, p = 0.62). Final model of logistic regression showed no association between serum Hcy and cognitive function after adjusting confounding variables (OR: 1.778; 95%CI: 0.69–4.54). Further investigation involving slight elderly T2DM patients with larger sample size should be conducted to confirm this finding.

## Introduction

Hyperglycemia, insulin resistance, and relative lack of insulin were key features of Type-2 Diabetes Mellitus (T2DM). Currently, there are 366 million people with diabetes mellitus worldwide and it will reach 552 million by 2030. T2DM has been associated with many chronic complications involving many organs, including brain and nervous system.[1–4] Epidemiological evidences suggest that T2DM is strongly associated with impaired cognitive function and structural abnormalities of brain.[2,5–10] Primary pathological mechanism of T2DM cognitive impairment is brain insulin resistance.[11,12] Ten years cohort study of 1617 non-dementia older participant showed an increased risk of dementia and mild cognitive impairment compared healthy control group.[13] A meta-analysis estimated that patients with T2DM had risk for vascular dementia (RR 2.5, 95% CI: 2.1–3.0) and Alzheimer disease (RR 1.5, 95% CI 1.2–1.8) compared to individuals without diabetes.[2] In structural abnormalities, T2DM is associated with total gray matter volume and also middle temporal gyrus (MTG) volume.[7]

One of the strongest risk factor for cognitive decline is age.[14] Studies showed that contributing factors associated with T2DM-related cognitive impairment included poor glycemic control, obesity, diabetic retinopathy, and many other factors.[8,15,16] Besides, plasma homocysteine was associated with an increased risk for cardiovascular events, cerebral artery stenosis and cognitive dysfunction independent of conventional risk factors.[17–21] Pathophysiologic mechanisms underlying those complications were the roles of homocysteine in promoting oxidative stress, inflammation, thrombosis, endothelial dysfunction, and cell proliferation.[19,22,23] Some studies showed a significant increased level of plasma homocysteine in T2DM patients.[24,25] However, there was a conflicting result occurred in non-DM population.[26] A case-control study involving T2DM patients aged 50–75 years old found that increased plasma homocysteine level was significantly associated with T2DM-related mild cognitive impairment (MCI), especially executive dysfunction.[27] This study aimed to investigate association between homocysteine level and cognitive impairment in middle aged T2DM patients.

## Materials and methods

### Design and ethics

**S**tudy was conducted in Endocrinology Clinic of Cipto Mangunkusumo National General Hospital, a tertiary care hospital in Jakarta–Indonesia. This was a cross-sectional study using primary data, involving <60 years old T2DM patients in outpatient setting. This study received ethical clearance from Ethical Committee of Faculty of Medicine, Universitas Indonesia—Cipto Mangunkusumo National General Hospital (number 0222/UN2.F1/ETIK/2018). Informed consents were obtained from all subjects in written.

### Sample size and eligibility

Sample size calculation was performed by applying mean difference of two independent groups formula, resulted 97 subjects. Subjects were diagnosed T2DM according to Indonesian national consensus of T2DM adapted from international guidelines.[28] Inclusion criteria were T2DM patients aged <60 years old with duration of diabetes ≥ 5 years recruited consecutively from June 2018 to October 2018, patients should be able to communicate verbally with doctor and able to read texts and pictures in the questionnaire. Exclusion criteria were any conditions affecting cognitive function and serum homocysteine level, including a history of central nervous system illness (stroke, CNS infection/malignancy/trauma, cerebral hemorrhage or surgery), end-stage kidney failure (stage IV-V CKD),[29,30] depression or psychosis, no experience of formal education,[31] cirrhosis,[32] and consuming drugs influencing serum homocysteine level (methotrexate, carbamazepine, phenytoin, nitrous oxide, 6-azauridine triacetate, folic acid or vitamin B_12_ supplementation).[33,34]

### Demography and clinical data collection

Demographic characteristics, including age, sex, education level, occupation, smoking history, exercise habit, and family history of diabetes were collected through a standardized interview. Education level was defined as duration of formal education starting from elementary school level in years. Exercise habit was defined as exercise conducted in a week; regular exercise suggested minimal 150 minutes of exercise per week divided in three sessions, while irregular exercise suggested less than that definition.[28,35] Clinical data including comorbidities, such as hypertension, dyslipidemia, heart disease, renal failure, neuropathy, and other vascular complication of T2DM, were documented. Medication histories, including oral anti-hyperglycemic drugs and used of insulin were documented for each subject. Physical examinations, including blood pressure and body mass index, were obtained using standard measurement. Body mass index (BMI) was defined as the body weight divided by the squared height [body weight (kg)/body height (m^2^)]. Diagnosis of hypertension, dyslipidemia, heart disease, renal failure and other T2DM complications were taken from medical record based on the criteria in an updated international guideline.[36,37] Subjects were asked to fast for at least in 6–8 hours before blood sample was taken. Blood samples were taken between 8–10 A.M to check the level of fasting blood-glucose, glycated hemoglobin, serum creatinine, triglyceride, total cholesterol, low density lipoprotein cholesterol (LDL-C), high density lipoprotein cholesterol (HDL-C), and serum homocysteine. Estimated glomerular filtration rate (eGFR) was calculated using Cockroft-Gault formula.

### Cognitive assessment

Montreal Cognitive Assessment is a highly sensitive screening tool for mild cognitive impairment in many conditions.[38–41] Cognitive functions of subjects were assessed using validated Indonesian version of Montreal Cognitive Assessment (MoCA-INA) consisting of 12 items cognitive domains, including short-term memory recall (5 points), visuospatial/executive function (5 points), attention-concentration-working memory (6 points), language (3 points), orientation to time and place (6 points), abstraction (2 points) and naming (3 points).[42] Maximum point of this test is 30 points. Global cognitive function was divided into 2 categories: mild cognitive impairment (MCI) if the score was <26, while normal score was ≥26. For subjects with education level less than 12 years, the score was corrected by addition of 1 point. An experienced neurologist from Department of Neurology, Cipto Mangunkusumo National General Hospital reviewed the test results. Subjects showed no audiovisual or motor coordination deficit affecting the test. Subjects with depression based on clinical examination and Beck Depression Inventory (BDI) were excluded.[31]

### Serum homocysteine level measurement

Venous blood samples were obtained and collected in anticoagulant-free tubes. The tubes were directly stored in 8°C box and transported to the central laboratory of hospital. Blood samples were centrifuged at 5000 rpm for 10 minutes to get serum. Serum was stored at -30°C refrigerator until sample size were achieved for simultaneous measurement. Quantitative measurement of serum homocysteine was performed using a chemiluminescent microparticle immunoassay (CMIA) method and Abbott Architect i2000 reagen kit. Normal range for homocysteine level according to reference is 5–15 μmol/L.[34]

### Data analysis

Value of α was set at 5%, while the statistical power was 90%. Normality data distribution was tested using Kolmogorov-Smirnov test. Mean and standard deviation was used to describe data in normal distribution, while median (minimum-maximum) was used to describe data in abnormal distribution. Mean difference of two groups was compared with independent t-test for normal data or Mann-Whitney test for abnormal data. Relationship between serum homocysteine and global cognitive function was analyzed using chi-square test. Bivariate analysis was performed for all covariates affecting relationship between homocysteine level and cognitive function: HbA1C, hypertension, dyslipidemia, BMI and exercise.[43–49] Multivariate analysis was performed with logistic regression model to assess confounding variables. Full model was made by selecting variables with p-value <0.25. Confounding test was performed with Backward LR method to determine confounding variables in association of serum homocysteine level and cognitive function. Statistical analysis was performed using SPSS version 20.0. Statistical significance was set in p-value of <0.05.

## Results

**Fig 1:**
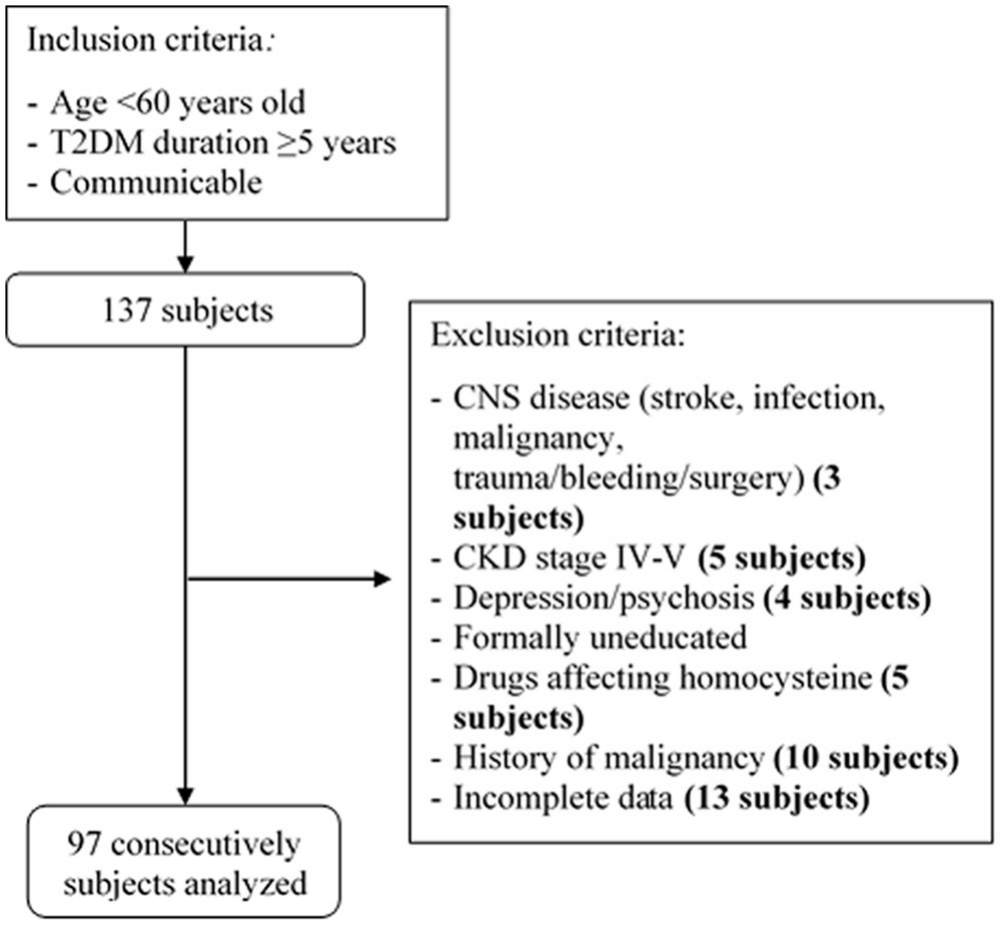
Flow chart of subject recruitment process.

In this study, there were 97 T2DM subjects based on flow-chart of subject recruitment process in Endocrinology outpatient clinic in Cipto Mangunkusumo National General Hospital (Fig 1).

Fig 1 showed flow-chart of subject recruitment process in Endocrinology outpatient clinic in Cipto Mangunkusumo National General Hospital. T2DM: Type 2 Diabetes Mellitus; CNS: Central Nervous System. CKD: Chronic Kidney Disease.

Demographic characteristics, clinical data and cognitive assessment of 97 subjects were documented. Table 1 showed similar demographic characteristics between impaired cognitive group and normal cognitive group except variable of diabetes duration, eGFR, LDL-C, post-prandial blood glucose and MoCA-INA score.

**Table 1:** Demographic and clinical characteristics of subjects based on cognitive function.

Variable	Cognitive Function	
	Impaired (n = 47)	Normal (n = 50)
Age (years)	54 (36–59)	54 (39–59)
Female (n (%))	29 (61.7%)	29 (60.4%)
Education (years)	12 (6–18)	13 (3–19)
Diabetes duration (years)	8 (5–21)	11 (5–26)
Systolic BP (mmHg)	130 (110–205)	130.5 (90–185)
Diastolic BP (mmHg)	80 (54–107)	80 (60–100)
Body mass index (kg/m^2^)	26.54 (4.79)	27.39 (4.25)
Glomerular filtration rate (ml/min)	75.70 (35.5–114)	81.75 (32.7–180.8)
Fasting blood glucose (mg/dl)	155 (84–446)	147 (64–390)
Post-prandial blood glucose (mg/dl)	249.43 (105.17)	204.09 (98.26)
HbA1C (%)	7.7 (5.6–13.3)	7.85 (4.8–12.9)
Total cholesterol (mg/dl)	201.23 (62.97)	202.54 (46.58)
HDL cholesterol (mg/dl)	46 (15–72)	47 (23–186)
LDL cholesterol (mg/dl)	129.89 (51.94)	135.06 (47.49)
Triglyceride (mg/dl)	153 (71–751)	151 63–536)
Homocysteine (mmol/L)	11.99 (3.29)	12.36 (4.07)
Hypertension (n (%))	42 (89.4%)	38 (77.6%)
Dyslipidemia (n (%))	44 (93.6%)	46 (93.9%)
Exercise (n (%))	9 (19.1%)	11 (22.4%)
MoCA-INA score	24 (20–25)	27 (26–30)
BDI score	7.89 (4.46)	7.20 (3.88)

Table 2 showed there were seven domains of cognitive impairment. Delayed recall was the most cognitive impaired domain (94.8%) while naming was the least cognitive impaired domain (10.3%).

**Table 2:** Distribution of cognitive impairment domain based on MoCA-INA test.

Domain	N (%)
Visuospatial/executive	46 (47.4)
Naming	10 (10.3)
Attention	35 (36.1)
Language	58 (59.8)
Abstraction	33 (34)
Delayed recall	92 (94.8)
Orientation	14 (14.4)

Table 3 showed mean serum homocysteine level in impaired cognitive group was 11.99 μmol/L (SD 3.29), while in normal cognitive group was 12.36 μmol/L (SD 4.07). Statistical analysis showed there was no significant mean difference of serum homocysteine level between two groups (p = 0.62).

**Table 3:** Serum homocysteine and cognitive function.

Variable	Cognitive function		p-value
	Impaired (n = 47)	Normal (n = 50)	
Homocysteine (μmol/L)	11.99 (3.27)	12.36 (4.07)	0.62

Significance p <0.05

Table 4 demonstrated association between confounding variables and cognitive function. There was similar result of HbA1C, dyslipidemia, and exercise variables between impaired cognitive group and normal cognitive group. Meanwhile, there was different result of hypertension and BMI variables between impaired cognitive group and normal cognitive group. Based on result, variables with p value <0.25 will further be included to multivariate analysis.

**Table 4:** Bivariate analysis of cognitive function and confounding variables.

Variable	Category	Cognitive function		Total	OR (95%CI)	p value
		Impaired	Normal			
HbA1C	Uncontrolled	31 (66%)	34 (68%)	65	0.91 (0.39–2.12)	0.83
	Controlled	16 (34%)	16 (32%)	32		
Hypertension	Yes	42 (89.4%)	38 (76%)	80	2.65 (0.85–8.22)	0.08
	No	5 (10.6%)	12 (24%)	17		
Dyslipidemia	Yes	44 (93.6%)	46 (92%)	90	1.27 (0.27–6.02)	0.75
	No	3 (6.4%)	4 (8%)	7		
BMI	Obese	27 (57.4%)	37 (74%)	64	0.47 (0.20–1.11)	0.08
	Non-obese	20 (42.6%)	13 (26%)	33		
Exercise	Irregular	38 (80.9%)	39 (78%)	77	1.19 (0.44–3.19)	0.72
	Regular	9 (19.1%)	11 (22%)	20		

Significance. P<0.05. BMI, body mass index; HbA1C, glycated hemoglobin; OR, odds ratio; CI, confidence interval.

Multivariate analysis was performed for covariates with p value <0.25 to assess each variable’s role in association of serum homocysteine level and cognitive function. Table 4 showed only two variables were included in multivariable analysis which were hypertension (*p* = 0.08) and BMI (*p* = 0.08). Table 5 showed that there was no association of homocysteine level on cognitive function in full model of multivariate analysis (OR 1.917; 95%CI: 0.726–5.061). Assessment of confounding was performed used Backward LR method with OR difference (ΔOR) >10% which was suggesting a covariate as a confounding while ΔOR <10% which was not suggesting a covariate as a confounding.

**Table 5:** Confounding assessment in association between homocysteine level and cognitive function.

Model	Independent Variable	OR	CI 95%	ΔOR (%)	Result
Model 1 Full model	Homocysteine	1.917	0.726–5.061	-	-
Model 2 Without hypertension	Homocysteine	1.778	0.696–4.545	7.25%	Not confounding
Model 3 Without BMI	Homocysteine	1.347	0.566–3.205	24.24%	Confounding

BMI, body mass index; OR, odds ratio; CI, confidence interval.

Table 6 showed final model of multivariate analysis. Final model showed only BMI as confounding in association between homocysteine level and cognitive function. In addition, serum homocysteine level did not have effect on cognitive function even after adjusting for confounding.

**Table 6:** Final model of multivariate analysis.

Variable	B	SE	Crude OR		Adjusted OR		p-value
			OR	95%CI	OR	95%CI	
**Independent variable**							
Homocysteine	0,57	0.47	1.91	0.72–5.06	1.77	0.69–4.54	0.22
**Covariate**							
BMI	0.91	0.46	2.82	1.08–7.31	2.49	0.99–6.22	0.05

Significance. P<0.05. BMI, body mass index; B, regression coefficient; SE, standard error; OR, odds ratio; CI, confidence interval.

## Discussion

Cognitive impairment problem in this study was important, there were 47 subjects (48.5%) had cognitive impairment even after excluding major factors contributing to cognitive decline such as age, history of stroke, intracranial bleeding, surgery, tumor and infection. This cognitive function was assessed with Indonesian version of MoCA (MoCA-INA) proven to be highly sensitive in detecting mild cognitive impairment. Tian et al [27] found similar result with more than 50% of subjects with cognitive impairment. Subjects of this study was different from previous one because we only include subjects younger than 60 years old. Previous studies involved elderly patients who was commonly related to age-associated cognitive decline.[14] This finding showed that even after we restrict subjects to younger than 60 years old, cognitive impairment was still an important issue among T2DM patients.

Our study suggested that serum homocysteine level did not increase in middle-aged T2DM patients. The mean of serum homocysteine level was 12.18 μmol/L (SD 3.69), was still lower than upper reference limit which was 15 μmol/L. Malaguarnera et al [50] also demonstrated similar mean homocysteine levels in T2DM subjects (n = 50) which was 12.1 μmol/L (SD 6.8). In contrast, those with proliferative diabetic retinopathy (n = 62) and non-proliferative diabetic retinopathy (n = 63) have mean homocysteine levels of 18.2 μmol/L (SD 5.6) and 14.4 μmol/L (SD 6.7), respectively. Besides, significant increased homocysteine level was found in 175 T2DM subjects with mean age 65.2 years (SD 11.8).

The result of this study was different from the previous study. A case series study conducted by Shaikh et al [24] involving 80 T2DM subjects <60 years old showed that 48 subjects (60%) were hyperhomocysteinemia. The study applied similar recruitment criteria regarding conditions affecting homocysteine level which were severe hepatic impairment, renal impairment, psoriasis, autoimmune disease, various malignancy conditions, supplementation of folic acid, pyridoxine and vitamin B_12_. This difference might be caused by different homocysteine measurement method and different nutritional status of subjects.[34] Majority of subjects (76%) recruited by Shaikh et al were from rural regions of India. Role of nutritional status need to be explored further in the future studies.

Our study did not find significant difference of serum homocysteine level between MCI and non-MCI group. This study involved middle-aged subjects <60 years old to investigate association between homocysteine and cognitive impairment in younger T2DM populations. In contrast, Tian et al [27] found mean serum homocysteine in MCI group was higher (14.28 μmol/L; SD 0.18) than non-MCI group (9.74 μmol/L; SD 0.18) with *p* <0.001. Although the mean of serum homocysteine level of two group in normal homocysteine concentration range (5–15 μmol/L), it was still clinically important and statistically significant. Their study involved subjects aged 50–75 years old with mean age of MCI subjects was 61.14±0.56 years old. One of the major factors contributing to hyperhomocysteinemia state is age.[12] Significant mean difference may be attributed to age-associated hyperhomocysteinemia. This hypothesis needs further investigation in the future studies.

Multivariate analysis result showed that BMI was confounding for association between serum homocysteine level and cognitive function. After adjusting BMI as a confounding in association, role of serum homocysteine on cognitive function was still not statistically significant (OR 1.778, CI95% 0.696–4.5454, *p* = 0.229). This result showed that serum homocysteine did not affect global cognitive function despite after adjusting confounding.

## Conclusion

This study showed consistent finding that T2DM strongly affect cognitive function. Serum homocysteine level was not associated with global cognitive function in middle-aged T2DM population. It might be due to younger age of subject influencing the low level of homocysteine. Further studies involving slight elderly T2DM patients with larger sample size were needed to investigate association between homocysteine level and cognitive function.

## Supporting information

S1 File This is data of the manuscript of association between serum homocysteine level and cognitive function in middle-aged type 2 diabetes mellitus patients. (XLS)
